# Factors and processes modulating phenotypes in neuronopathic lysosomal storage diseases

**DOI:** 10.1007/s11011-013-9455-6

**Published:** 2013-12-05

**Authors:** Joanna Jakóbkiewicz-Banecka, Magdalena Gabig-Cimińska, Zyta Banecka-Majkutewicz, Bogdan Banecki, Alicja Węgrzyn, Grzegorz Węgrzyn

**Affiliations:** 1 Department of Molecular Biology, University of Gdańsk, Wita Stwosza 59, 80-308 Gdańsk, Poland; 2 Laboratory of Molecular Biology, Gdańsk University, Institute of Biochemistry and Biophysics, Polish Academy of Sciences, Wita Stwosza 59, 80-308 Gdańsk, Poland; 3 Department of Neurology, Medical University of Gdańsk, Dębinki 7, 80-211 Gdańsk, Poland; 4 Intercollegiate Faculty of Biotechnology, University of Gdańsk and Medical University of Gdańsk, Kładki 24, 80-822 Gdańsk, Poland; 5 Department of Microbiology, University of Szczecin, Felczaka 3c, 71-412 Szczecin, Poland

**Keywords:** Inherited metabolic diseases, Lysosomal storage diseases, Neuronopathic forms of heritable disorders, Genotype-phenotype correlations, Factors modulating disease severity

## Abstract

Lysosomal storage diseases are inherited metabolic disorders caused by genetic defects causing deficiency of various lysosomal proteins, and resultant accumulation of non-degraded compounds. They are multisystemic diseases, and in most of them (>70 %) severe brain dysfunctions are evident. However, expression of various phenotypes in particular diseases is extremely variable, from non-neuronopathic to severely neurodegenerative in the deficiency of the same enzyme. Although all lysosomal storage diseases are monogenic, clear genotype-phenotype correlations occur only in some cases. In this article, we present an overview on various factors and processes, both general and specific for certain disorders, that can significantly modulate expression of phenotypes in these diseases. On the basis of recent reports describing studies on both animal models and clinical data, we propose a hypothesis that efficiency of production of compounds that cannot be degraded due to enzyme deficiency might be especially important in modulation of phenotypes of patients suffering from lysosomal storage diseases.

## Introduction

There are several thousand inherited diseases identified to date (Korf and Rehm 2013), among which many affect brain functions. An example is a group of metabolic disorders caused by genetically determined dysfunctions of enzymes or non-enzymatic proteins required for functions of lysosomes, known as lysosomal storage diseases or LSDs (for a recent review see Platt et al. 2012). All LSDs are monogenic disorders, and each of them is characterized by deficiency in activity of one of polypeptides involved in degradation of particular macromolecular compounds or in other lysosomal functions. Hence, accumulation of certain undegraded compound(s) occurs in each LSD, and due to continuous synthesis of these compounds, the disease has a progressive character. In fact, clinical symptoms of some LSDs appear as late as at the age of a few or even several years, but then they become more and more severe. LSDs are classified on the basis of the nature of accumulated compound(s) and the kind of deficient enzyme, encoded by a corresponding gene. Thus, over 50 types and subtypes of LSDs are currently recognized (reviewed by Cox and Cachon-Gonzalez 2012). It is worth noting that – contrary to early hypotheses – despite the fact that development of each LSD depends on dysfunction (or deletion) of particular gene and resultant deficiency of activity of particular gene product, it is not obvious whether primary storage of various materials are primary causes of the LSDs.

LSDs are amongst the most commonly confirmed diagnoses of neurodegeneration, reaching as many as 45 % of cases (Verity et al. 2010). In fact, most of LSD types (>70 %) result in severe dysfunctions of central nervous system, while patients suffering from other LSDs may be non-neuronopathic or their neurological symptoms may be mild (reviewed by Jardim et al. 2010; Arfi et al. 2012). Although neuronopathy depends on insufficient degradation of certain compounds, it is still unclear whether symptoms resulting from central nervous system involvement are due to storage material in extracellular matrix or abnormality of the extracellular matrix itself (as effects of reactions secondary to the storage), or both. For instance, severity of neurodegenerative sphingolipidoses may be associated to changes of the properties of plasma membranes, including trafficking defects of membrane lipids and proteins (Eckhardt 2010). Another interesting example is Gaucher disease, the most common LSD, which is caused by dysfunction of glucocerebrosidase and resultant accumulation of glucocerebroside that cannot be cleaved to glucose and ceramide (Sidransky 2004). A clinical link between this disease and Parkinson disease has been described, which was expressed as relatively early symptoms of parkinsonism in Gaucher disease patients (Sidransky 2005). This suggested that mutations in the *GBA1* gene (coding for the glucocerebrosidase), and resultant alterations in sphingolipid metabolism, may contribute to biochemical changes found in Parkinson disease. More recent studies indicated that a lack of the glucocerebrosidase activity compromises lysosomal protein degradation and causes accumulation of α-synuclein, which results in neurotoxicity (Mazzulli et al. 2011). In fact, appearance of aggregates of this protein is characteristic for Parkinson disease neurons (for a review, see Olanow and Brundin 2013).

Despite our incomplete understanding of the primary cause(s) of cell, tissue and organ dysfunctions in LSD, it is well documented that clinical symptoms arising from accumulation of incomplete degradation of the same substance which hydrolysis is inhibited at various stages can be significantly different. For example, brain disease in patients suffering from both mucopolysaccharidosis type I (MPS I) and mucopolysaccharidosis type III (subtypes A, B, C and D) (MPS III subtypes A to D) results from accumulation of metabolites of heparan sulfate (HS), one of glycosaminoglycans (GAGs) (for a recent review, see Muenzer 2011). However, in MPS I patients with neuronopathy, despite problems with learning and cognitive deterioration, little or no behavioral problems occur (reviewed by Campos and Monaga 2012). These patients are usually placid, gentle, calm, and often over-careful, even if cognitive deterioration is severe. On the other hand, MPS III patients (all subtypes), develop an aggressive behavior, they are hyperactive, and have usually severe sleeping defects. Moreover, these patients appear to ignore any danger, and their behavior suggests intensive action without any particular sense (discussed by Valstar et al. 2008; Wijburg et al. 2013). It was suggested that certain chemical moieties occurring at the ends of incompletely degraded HS molecules may determine characteristic behavioral disturbances, perhaps due to chemical reactions interfering with functions of neurons in the brain (Wegrzyn et al. 2010). In fact, HS degradation is inhibited at various stages in patients suffering from MPS I and MPS III A-D, and thus, different metabolites accumulate in each of these diseases.

Interestingly, there is a huge variability in patients’ phenotypes not only between different LSD, but also between different patients suffering from the same type of disease. A classical example is MPS I, mentioned above. This disease is caused by mutations in the *IDUA* gene, coding for α-L-iduronidase, and resulting in lysosomal storage of two GAGs: HS and dermatan sulfate (DS). The spectrum of neuronopathic symptoms of this disease is from severe mental and cognitive disability to completely normal intelligence and behavior, including all intermediate phenotypes (note that somatic symptoms occur in all MPS I patients, but with different severity). In fact, deficiency of α-L-iduronidase has been initially described as two different diseases, Hurler disease (MPS I) and Scheie disease (mucopolysaccharidosis type V or MPS V). Subsequent studies demonstrated that both disorders are caused by mutations in the same gene, *IDUA*, and led to the conclusion that they are just two extreme clinical phenotypes of the same metabolic disease (summarized by Lashford et al. 1999).

The obvious question appearing in the light of the above mentioned variability in clinical spectra of one disease is: what is responsible for such big differences in phenotypes of patients suffering from dysfunction of just one gene? Perhaps surprisingly, we are still not able to answer this question precisely, despite unquestionable advance in understanding molecular, cellular and physiological mechanisms of LSDs. In this article, we will summarize our current knowledge on genotype-phenotype correlations in these diseases as well as physiological and environmental factors modulating effects of mutations in single genes.

## Genotype-phenotype correlations in LSDs

Determination of a genotype-phenotype correlation is the primary approach in attempts to understand variability of any monogenic disease, and to predict the level of symptoms’ severities if early diagnosis was made (Lyonnet et al. 2006). Therefore, it is not a surprise that many articles were published in which authors tried to understand genotype-phenotype correlations in different inherited metabolic diseases, including LSDs. However, contrary to an early assumption that knowing mutation(s) in the affected gene one should be able to predict patient’s phenotype (highlighted and discussed by Silverman and Mahadevan 2005), after years of studies, it appears that such a scenario is possible only in some cases, and often the clinical phenotype is weakly related to the particular mutation (reviewed by Lippi and Favaloro 2009). In LSDs, a general scheme can be presented that non-sense mutations or other mutations that result is a complete lack of activity of corresponding genes’ products (like most of deletions, insertions, and other types of gene rearrangements), called *null* mutations, cause severe phenotypes (for a review and discussion, see Filocamo and Morrone 2011). This is also true for diseases in which neuronopathy occurs only in a fraction of patients, like MPS I or Gaucher disease. In these cases, *null* mutations cause a neuronopathic form of the disease, while mutations allowing an appearance of a residual enzyme activity (like missense mutations) may result in attenuated, non-neuronopathic phenotypes (Terlato and Cox 2003; Vitner and Futerman 2013). Nevertheless, while the former rule is usually true, the latter one is often false, as there are many examples of severe neuronopathic phenotypes of patients bearing missense mutations (among many reports on this topic, see for example, Chkioua et al. 2011; Michelakakis et al. 2006).

There are many reports in the literature describing large problems in finding unambiguous genotype-phenotype correlations in LSDs. Below, we will discuss only very few of them, just to exemplify the level of complication of the problem.

Fabry disease is an X-linked LSD, in which mutations in a gene coding for α-galactosidase A (α-GAL) result in impaired degradation of certain glycoconjugates and accumulation of globotriaosylceramide (Gb3) in various tissues including kidneys, heart, and the nervous system. As discussed by Ries and Gal (2006), there is a high degree of clinical variability both among patients from the same family and among those from unrelated families with the same mutation. Therefore, in this disease, knowing the mutation type in the α-GAL gene is of relatively little help for prediction of patient’s phenotype. Moreover, since the same genotype can result in different phenotypes, it is clear that other factors must influence the disease expression.

There are 3 clinical forms of Gaucher disease (glucocerebrosidase deficiency): type 1 is restricted to visceral and/or skeletal involvement with no brain dysfunction, type 2 is a neuronopathic form characterized by severe central nervous system involvement in infancy, and type 3 is an intermediate form, with milder neuronopathy. In Gaucher disease, some clear genotype-phenotype correlations could be detected. The N370S mutation (in either homozygotic or complex heterozygotic form) is restricted to patients with type 1 disease, while homozygosity for the L444P mutation is highly associated with type 3 disease (Koprivica et al. 2000). However, among L444P homozygous patients, a high variability in phenotypes was reported, though all were classified within type 3 (Goker-Alpan et al. 2005). Another mutation, R463C, was found in patients with type 1 as well as with type 3 disease (Koprivica et al. 2000). Analysis of many kinds of mutations in the glucocerebrosidase gene of patients suffering from various types of Gaucher disease revealed a considerable genotypic heterogeneity among clinically similar patients, and significantly different phenotypes among patients with the same mutations (Sidransky 2004). An extremely interesting case of monozygotic twin sisters has been reported by Biegstraaten et al. (2011). These sisters, both being homozygous for the N188S mutation, expressed extremely different phenotypes of Gaucher disease. One of them had severe visceral involvement, epilepsy, and a cerebellar syndrome, while the second developed only type 1 diabetes mellitus with no other symptoms of Gaucher disease. Equally intriguing observations were reported earlier by Lachmann et al. (2004), where two pairs of twins have been described. In the first pair, from two monozygotic sisters homozygous for the N370S mutation, only one developed Gaucher disease symptoms while the second remained asymptomatic for this disease until she died at the age of 84 years. The second pair consisted of dizygotic twins being compound heterozygotes for the N370S and L444P alleles, from which only one has developed Gaucher disease symptoms by the age of 57 years. All the results mentioned in this paragraph indicate that although some genotype-phenotype correlations are evident in Gaucher disease, there are many mutations which effects must be modified by other factors to result in such a variability of patients’ phenotypes.

Mutations in both alleles of the *GAA* gene, coding for acid α-glucosidase, result in accumulation of glycogen and clinical symptoms described as Pompe disease. Variability of phenotypes is large in this disease, from a very severe infantile form to more attenuated ones which clinical expression may occur in adults. Genotype-phenotype correlation in Pompe disease is quite strict relative to some other LSDs, as the severe infantile form is restricted to mutations leading to a total lack of *GAA* expression or a complete lack of produced acid α-glucosidase (Kroos et al. 2012) in both alleles. However, some other genotypes, like c.-32-13 T > G/*null*, result in very different phenotypes of patients (Kroos et al. 2012).

Mucopolysaccharidosis type VI (MPS VI) is a disorder caused by deficiency in N-acetylgalactosamine 4-sulfatase (arylsulfatase B) activity due to mutations in the *ARSB* gene. This leads to the storage of dermatan sulfate (DS), one of GAGs. Amongst over 130 pathogenic mutations described in the *ARSB* gene, most are missense mutations (Saito et al. 2012). Despite the fact that some genotype-phenotype correlations have been clear, our understanding of effects of particular changes in the sequence of *ARSB* on the disease severity is far from being complete (Valayannopoulos et al. 2010; Brands et al. 2013). Interestingly, although MPS VI usually affects many organs, a specific, predominantly cardiac, phenotype has been described recently, which is associated with the homozygous R152W mutation in the *ARSB* gene (Jurecka et al. 2011, 2013). However, molecular mechanism of such an expression of this disease in R152W/ R152W patients is unknown.

In summary, while some genotype-phenotype relationships are evident in LSDs, there are plenty of examples that the same mutation may result in very different clinical symptoms and different progress of the disease. Moreover, level of residual activity of the deficient enzyme often does not correspond to the disease severity.

## General physiological processes and environmental factors influencing LSDs

Since genotype-phenotype correlations cannot be determined in many cases of LSDs, it became clear that other factors or processes must influence severity of symptoms occurring in particular patients. In fact, publications from last several years indicated that there are various physiological processes and environmental factors that can significantly modify the course of LSDs. Since mechanisms of these processes and actions of these factors have been excellently reviewed in past few years, we will present them only briefly in this overview, describing their crucial features and refereeing readers to previously published articles for details.

Contrary to early assumptions on the mechanisms of LSDs, it is now obvious that various symptoms appear not only due to primary storage of undegraded compounds in lysosomes (in fact, the primary lysosomal storage has been questioned as the primary cause of these diseases; Platt et al. 2012), but also because of secondary and tertiary effects. The primary storage may affect activities of various lysosomal proteins, thus, deficiency in degradation of different compounds and their secondary accumulation can occur. This, in turn, leads to further disturbances in cell physiology, including activation of some receptors by ligands that cannot do this under physiological conditions, changes in receptors’ responses to specific mediators, and modifications of signal transduction cascades due to changes in functions of intracellular effectors (for a review, see Ballabio and Gieselmann 2009). Then, various cellular stress responses are activated, but due to continuous and progressive accumulation of the primary and secondary storage compounds they cannot restore the physiological balance and rescue the cells. Thus, not only cellular but also systemic reactions are involved in the disease pathomechanism. The pathological effects occurring at different levels of the organism organization, include, but are not restricted to, oxidative stress, endoplasmic reticulum stress, defects in autophagy, dysfunction of the Golgi apparatus, epigenetic factors, mitochondrial dysfunction, peroxisomal dysfunction, altered calcium homeostasis, abnormal trafficking of various compounds, inflammatory processes, autoimmune response, and energy imbalance. These LSD-associated effects have been described and discussed recently by other authors (Bellettato and Scarpa 2010; Parkinson-Lawrence et al. 2010; Vitner et al. 2010; Filocamo and Morrone 2011; Hawkins-Salsbury et al. 2011; Schultz et al. 2011; Cox and Cachon-Gonzalez 2012; Platt et al. 2012), therefore they will not be reviewed in detail here.

It is obvious that different intensities of the above mentioned processes may significantly influence the phenotypes of patients suffering from LSDs, thus modulating severity of the disease. Moreover, external factors may contribute to expression of the specific processes and responses. This may be exemplified by microbial infections, which are more often in LSD patients than in general population due to impaired immunological reactions, changed cellular physiology and others. Such an influence was described, for instance, in MPS I, where atypical microbial infections of digestive tract contributed considerably to severity of the disease, causing frequent diarrhea and worsening of a general patient status (Wegrzyn et al. 2005).

## Specific processes affecting severity of particular LSDs

Although physiological processes and environmental factors described in the preceding chapter may significantly influence severities of various LSDs indeed, it is rather unlikely that they are the only determinants responsible for different phenotypes of patients bearing the same mutations, including siblings. If such a scenario were true, it should be possible to manage the disease at the level of the attenuated form by controlling these processes and factors, and very similar severity of the disease should be observed among siblings that bear the same mutation(s) and live together under very similar conditions, which is definitely not the case. Therefore, one may assume that there are processes specific to each disease which can significantly modulate its course and severity.

Vast majority of LSDs results from an imbalance between synthesis and degradation of particular compounds. While the synthesis process is unaffected, the degradation is impaired which leads to accumulation of certain substances in lysosomes. About a decade ago it was speculated that various efficiencies of syntheses of particular compounds, which may occur in human population, could significantly influence severity of LSDs and result in different phenotypes of patients bearing the same mutations in genes responsible for degradation of these compounds (Wegrzyn et al. 2004). More recent studies on mechanisms of LSDs, described below, suggested that this hypothesis may be potentially true.

Metachromatic leukodystrophy (MLD) is caused by deficiency in arylsulfatase A (ASA) and accumulation of sulfated glycosphingolipids, predominantly 3-*O*-sulfogalactosylceramide (sulfatide). Neuronopathy of this disease is primarily due to demyelination of neurons. To learn about molecular mechanisms of this disease, a mouse model was constructed in which a homologue of the human *Arsa* gene, coding for ASA, has been inactivated (Hess et al. 1996). Surprisingly, mice homozygous for the knock-out (*null*) mutation (ASA^-/-^) did not reveal demyelination, and thus, characteristic symptoms of human MLD, like reduction in nerve conduction velocity, progressive paralysis, and peripheral neuropathy were not observed in these mice (Wittke et al. 2004). Such a model appeared to be of a limited usage in studies on mechanisms of the human disease, therefore, further work was necessary to make affected mice more similar to patients suffering from MLD. It was demonstrated that overexpression of the gene coding for galactose-3-*O*-sulfotransferase-1, an enzyme responsible for production of sulfatide, resulted in effective demyelination of mouse neurons and appearance of symptoms resembling those observed in the human disease (Ramakrishnan et al. 2007). Interestingly, synthesis of sulfatide in the galactose-3-*O*-sulfotransferase-1-overproducing ASA^-/-^ mice was only about 2–fold higher than that in the ASA^-/-^ mutant (Ramakrishnan et al. 2007; Matthes et al. 2012). This indicated that relatively minor changes in efficiency of synthesis of a particular compound may significantly influence severity of a lysosomal disease which arises from inefficient degradation of this compound (substrate), including appearance of neuronopathic symptoms when the synthesis is elevated.

Contrary to stimulation of synthesis of the substrate that cannot be degraded in lysosomes, partial inhibition of production of another substrate (HS), accumulated in: MPS IIIA and MPS IIIB, caused improvement of biochemical parameters and phenotypes of affected animals. This was demonstrated by chemical impairment of HS synthesis by the use of either rhodamine B ([9-(2-carboxyphenyl)-6-diethylamino-3-xanthenylidene]-diethylammonium chloride) (Roberts et al. 2006, 2007) or genistein (5, 7-dihydroxy-3- (4-hydroxyphenyl)-4*H*-1-benzopyran-4-one) (Malinowska et al. 2009, 2010). In those studies, biochemical parameters of MPS IIIA and MPS IIIB mice were improved in treated animals relative to untreated ones, including a decreased HS storage in brain. Moreover, behavior of these animals, severely affected in the absence of treatment, could be improved (Roberts et al. 2007) or even corrected (Malinowska et al. 2010). It was proposed that putative mechanisms of reduction of HS storage due to impairment of its synthesis may include dilution of already accumulated molecules in the processes of cell growth and division, and action of endoglycosidases, like heparanase, in combination with functional hydrolases operating at non-inhibited steps of the degradation (Banecka-Majkutewicz et al. 2012).

Although the experiments with chemically impaired synthesis of the substrate production might corroborate the conclusions made on the basis of studies on MLD animals, it could not be excluded that the used compounds, rhodamine B and/or genistein, might modify other processes, like oxidative stress or inflammation. In this light, it is crucial to note that a very interesting study on the MPS IIIA mouse model has been reported recently (Lamanna et al. 2012). In this model, the *Sgsh* gene, coding for sulfamidase, an enzyme required for degradation of HS, was mutated. Such MPS IIIA mice developed symptoms similar to those found in the human disease, including severe neuronopathy. These symptoms could be significantly attenuated when animals were also heterozygotic for additional mutation(s) in one or two genes coding for enzyme(s) involved in HS synthesis, Ext1 and Ext2, which resulted in at most 2-fold decrease in the rate of production of this GAG. Therefore, reduction of HS by 30–50 % led to significant decrease in severity of the disease, including amelioration of the amount of disease-specific biomarker in the brain (Lamanna et al. 2012).

The above described studies on mouse models of LSDs, MPS IIIA and MPS IIIB, strongly suggested that expression of specific symptoms of various diseases, including neuronopathy, depends not only on dysfunction of an enzyme involved in degradation of certain compound(s), but also on efficiency of production of the substrate(s) that cannot be efficiently degraded in lysosomes. More specifically, less efficient production should result in less severe symptoms. Do we have any evidence that similar phenomena may occur in humans suffering from LSDs? A few years ago, a report was published in which efficiency of GAG synthesis was measured in fibroblasts of healthy persons and patients suffering from various types of MPS (Piotrowska et al. 2009). In both groups (healthy persons and MPS patients), a considerable variability in kinetics of GAG production was observed, with the differences between the lowest and highest values determined for different subjects as high as 10 times. Therefore, it appears that a high natural variability in efficiency of GAG synthesis occurs in the natural human population. This has minor or negligible effects on physiology of persons with fully functional lysosomal system, however, it may significantly influence severity of a disease arising from impaired GAG degradation. In fact, MPS patients with low efficiency of GAG synthesis had milder phenotypes than those producing GAGs more efficiently, provided that detectable residual activity of the deficient enzyme involved in GAG degradation was found (Piotrowska et al. 2009). Therefore, one may speculate that the hypothesis made on the basis on studies on animal models might be also valid for humans. Obviously, there is a question whether the hypothesis based on the examples provided above, and concerning particular diseases, can be extrapolated to other LSDs? For example, to our knowledge, there are no published results demonstrating a role for biosynthetic pathways in final levels of the storage in patients suffering from sphingolipidoses, a group of LSD (see recent review by Schuchman and Simonaro 2013). Thus, further testing of the presented hypothesis is deserved.

## Concluding remarks

**Fig. 1:**
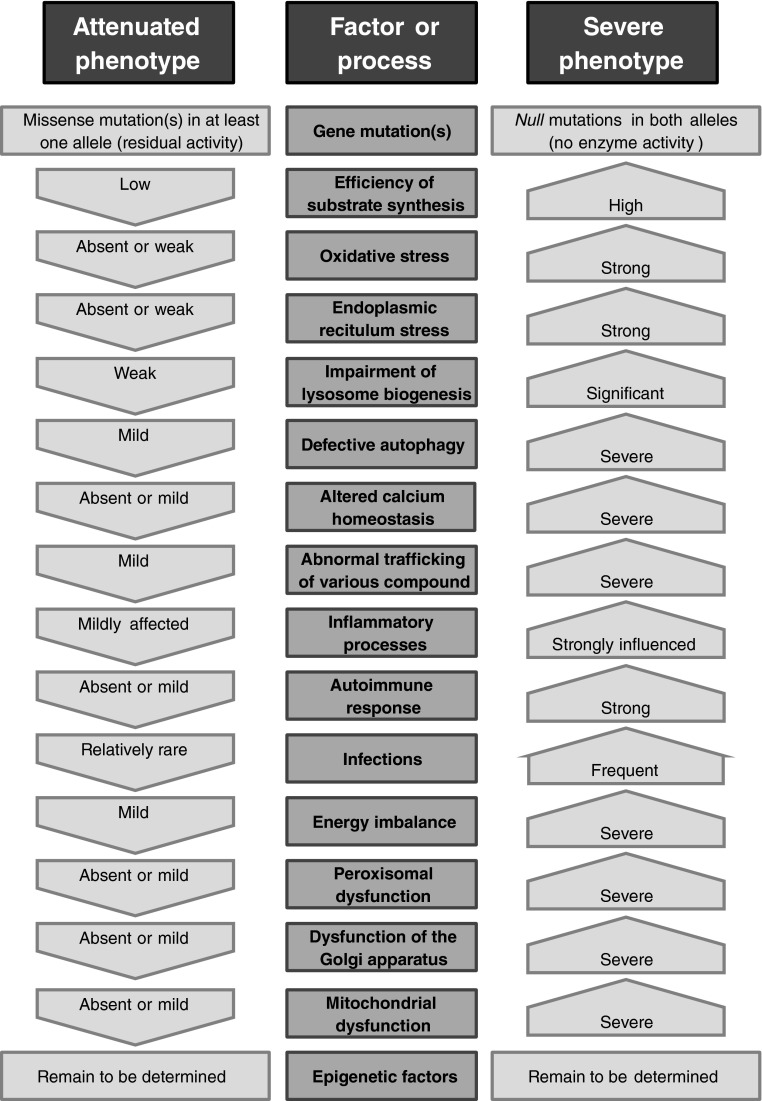
Factors and processes determining severity of LSDs. The kinds of factors and processes are presented in the central column, while their features supporting attenuated or severe phenotypes are characterized in the left and right columns, respectively. Note that the order of appearance of particular factors on the scheme does not correspond to their importance, since currently it is not possible to determine which factors are more or less important in disease development

Genotype-phenotype correlations in LSDs are clear for some types of mutations, particularly when *null* mutations occur in both alleles of the affected gene (or in the only allele in males suffering from X-linked LSDs). However, phenotypes of patients bearing mutations resulting in residual activities of affected enzymes depend on other factors, particularly various stresses caused by different agents (e.g. oxidative stress, endoplasmic reticulum stress, defects in autophagy, altered calcium homeostasis, abnormal trafficking of various compound, inflammatory processes, autoimmune response, and energy imbalance). The summary on factors and processes determining severity of LSDs is presented schematically in Fig. 1. In fact, currently we are not able to precisely determine the level of importance of particular factors or processes in disease development. Nevertheless, it is tempting to speculate that kinetics of synthesis of substrates that cannot be efficiently degraded may be of special importance for expression of disease symptoms, especially neuronopathic ones. Since a high variability in the levels of synthesis of at least some lysosomally-degraded compounds occurs in human population, we propose a hypothesis that this parameter may be of special importance in determination of phenotypes of a large group of patients suffering from LSDs. However, since experimental data leading to this hypothesis are available only for some of LSDs, it remains to be elucidated whether similar mechanisms might operate in other diseases from this group.
